# SMOQ: a tool for predicting the absolute residue-specific quality of a single protein model with support vector machines

**DOI:** 10.1186/1471-2105-15-120

**Published:** 2014-04-28

**Authors:** Renzhi Cao, Zheng Wang, Yiheng Wang, Jianlin Cheng

**Affiliations:** 1 Department of Computer Science, Informatics Institute, Christopher S. Bond Life Science Center, University of Missouri, Columbia, MO 65211, USA; 2 School of Computing, University of Southern Mississippi, Hattiesburg, MS 39406-0001, USA

## Abstract

**Background:**

It is important to predict the quality of a protein structural model before its native structure is known. The method that can predict the absolute local quality of individual residues in a single protein model is rare, yet particularly needed for using, ranking and refining protein models.

**Results:**

We developed a machine learning tool (SMOQ) that can predict the distance deviation of each residue in a single protein model. SMOQ uses support vector machines (SVM) with protein sequence and structural features (i.e. basic feature set), including amino acid sequence, secondary structures, solvent accessibilities, and residue-residue contacts to make predictions. We also trained a SVM model with two new additional features (profiles and SOV scores) on 20 CASP8 targets and found that including them can only improve the performance when real deviations between native and model are higher than 5Å. The SMOQ tool finally released uses the basic feature set trained on 85 CASP8 targets. Moreover, SMOQ implemented a way to convert predicted local quality scores into a global quality score. SMOQ was tested on the 84 CASP9 single-domain targets. The average difference between the residue-specific distance deviation predicted by our method and the actual distance deviation on the test data is 2.637Å. The global quality prediction accuracy of the tool is comparable to other good tools on the same benchmark.

**Conclusion:**

SMOQ is a useful tool for protein single model quality assessment. Its source code and executable are available at: http://sysbio.rnet.missouri.edu/multicom_toolbox/.

## Background

With the development of many techniques and tools for protein tertiary structure prediction, a large number of tertiary structure models can be generated for a protein on a computer at a much faster speed than the experimental methods such as X-ray crystallography and nuclear magnetic resonance (NMR) spectroscopy [1,2]. It is becoming increasingly important to develop model quality assessment programs that can predict the qualities of protein models before their corresponding native structures are known, which can help identify quality models or model regions and guide the proper usage of the models [3]. Therefore, the last few rounds of CASP (Critical Assessment of Techniques for Protein Structure Prediction) experiments [4-6] dedicated one model quality assessment (QA) category to specifically evaluate the performances of protein model quality assessment methods, which stimulated the development of such methods and programs in the last several years.

Model quality assessment programs can be categorized into *clustering*-based methods [7-14], *single-model* methods [14-18], and hybrid methods [19,20] that combine the previous two. *Clustering* methods need a set of protein models associated with the same protein sequence as input and can output the *relative* quality scores by pairwise structural comparison (alignments). *Single-model* methods only need one model as input and can output the either *relative* or *absolute* qualities of the model. In general, clustering-based methods usually had better performances than *single-model* methods [6,20-22] in the past CASP experiments. However, clustering methods are highly dependent on the size and the quality distribution of the input models. It is hard for them to pick up best models in most cases, especially if the best model is not the average model that is most similar to other models. Therefore, it is increasingly important to develop single-model methods that can predict the quality of a single model without referring to any other models.

Model quality assessment programs can either output *global* quality scores [11,14,18,23] or *local* quality scores [20,24-27]. A global quality score measures the overall quality of an entire model, whereas local quality scores consisting of a series of scores, one for each residue, measure the quality of the positions of individual residues. For instance, a local quality score may be the predicted distance between the position of residue in a model and that in the native structure after they are superimposed. Because local quality assessment methods can predict residue-specific qualities, it can help identify regions of good quality that can be used or regions of poor quality that needed to be further refined.

Although local quality predictions are very useful, not many local quality assessment methods have been developed. The existent local quality assessment methods mostly use statistical structural environment profiles [26,28-31], energy potentials [32], or pairwise clustering techniques that output *relative* local qualities [19,33,34]. Verify3D [29,35] is a representative method that compares the structural environment of a query model of a protein with the expected structural profiles for the protein compiled from native protein structures in order to predict the quality of the model. The information that Verify3D used to generate statistical profiles includes secondary structure, solvent accessibility, and residue polarity. ProQres [36] is a machine learning method that uses the structural features calculated from the model with artificial neural networks to predict absolute local qualities.

In this work, we developed and extensively tested a machine learning software tool (SMOQ) that implements a local quality assessment method predicting the *absolute local* qualities of a single protein model [14]. SMOQ also uses structural features including secondary structure, solvent accessibilities, and residue contact information as input. However, different with Verify3D that directly evaluates the fitness of the structural features parsed from a model, SMOQ compares the structural features parsed from the model with the ones predicted from sequence, and uses the comparison results as input features. In addition to using the features briefly introduced in [14], we tested the effectiveness of new features such as sequence profiles and SOV scores [37] and trained support vector machines on a larger dataset (CASP8) to make predictions. Furthermore, we developed and benchmarked a new method to convert predicted local qualities into a global quality score. Our experiment demonstrated that the global quality scores converted from local quality scores were useful for assessing protein models, particularly the models of hard *ab initio* targets.

## Implementation

### Features for support vector machines (SVM)

We developed and tested three SVM-based predictors using basic, profile, and profile+SOV feature sets respectively. The features in the basic feature set include amino acid sequence, secondary structures, solvent accessibility, and residue-residue contacts. The profile feature dataset is the same as the basic feature set except that amino acid sequence was replaced with sequence profile generated from PSI-BLAST [38]. Compared with the profile feature set, the profile+SOV feature sets added as a feature the SOV (segment overlap measure of secondary structure) scores [37] between the secondary structures predicted from protein sequence and secondary structures parsed from model.

A 15-residue window centered on a target residue in a protein was used to extract features. 20 binary numbers represent an amino acid at each position in the window. We used software SSPRO [39] to predict the secondary structures and solvent accessibility based on the amino acid sequence parsed from each protein model. For each residue position within the window, the predicted secondary structure and relative solvent accessibility were compared with the ones parsed from the protein model by the software DSSP [40]. If they are the same, 1 will be input as a feature for secondary structure or relative solvent accessibility, respectively, otherwise 0.

We used NNcon [41] to predict the residue-specific contact probability matrix from a protein sequence. For each residue within the 15-residue sliding window, we first used DSSP to parse their coordinates in the models to identify the other residues that are >=6 residues away in the sequence and are spatially in contact (<=8Å) with the residue. And then we calculated their average predicted probabilities of being contact with the residue according to the contact probability matrix. This averaged value was used as a feature. We calculated the SOV score between the secondary structures predicted from sequence and the secondary structure parsed from model and used it as a feature according to the same approach in [37].

The input features in a window centered at a target residue in a model are used by SVMs to predict the distance deviation between the position of the residue in the model and that in the corresponding native structure. The larger the distance deviation, the lower is the local quality.

### Training data set

Our first training data set contains the complete tertiary structure models of 85 single-domain CASP8 targets (http://predictioncenter.org/casp8/domain_definition.cgi). These targets contain all the single-domain “template based modeling” (53 TBM targets), “template based modeling-high accuracy” (28 TBM-HA targets), “free modeling” (2 FM targets), “free modeling or template based modeling” (2 FM/TBM) targets.

Descriptions about the domain classifications can be found from CASP website (http://predictioncenter.org/casp8/doc/Target_classification_1.html). For each of these targets, only the first Tertiary Structure (TS) model for a TS predictor was included in our training dataset. These models generated about 600,000 training examples (i.e. feature-distance pairs for the residues in these models) in total. This data set was used to optimize the parameters of the Radial Basis Function (RBF) kernel used with our support vector machines (SVM). A SVM model of using the basic feature set was then trained on this data set using the optimized parameters before being tested on the test data set.

To fairly compare the performances of basic, profile, and profile+SOV feature sets, we also trained them on the same set generated from the protein models associated with the same 20 CASP8 targets. These 20 CASP8 single-domain targets also contain FM, TBM, and TBM-HA targets in a balanced way.

All of the training and testing targets are deliberately chosen to be single-domain proteins. This is because directly superimposing multi-domain model with its native structure often over estimates the distance deviations of residues in individual domains due to possible deviations in domain orientations. An alternative way would be to cut multi-domain models into individual ones and align each domain with its native structure. Since we have a reasonable number of single-domain targets of different modeling difficulty (i.e., TBM, TBM-HA, and FM), we have chosen to only use single-domain targets for training and testing.

### Training and cross-validation

The support vector machine tool SVM-light (http://svmlight.joachims.org/) was trained on the data set extracted from the CASP8 tertiary structure models. We applied several rounds of 5-folds cross-validation on the training data set. Each round used a different combination of parameters: -c “trade-off between training error and margin”, -w “epsilon width of tube for regression”, and –g “the gamma parameter in the RBF kernel”. The parameter combination that achieved the best performance in a 5-fold cross-validation was finally used to train a SVM model with all the training examples.

### Test dataset

In total, 84 CASP9 single-domain targets were used to blindly benchmark the performances of the QA tools. The tools were tested only using the first TS (tertiary structure prediction) model for each target. Partial TS models that did not have coordinates for all the residues were discarded. In total, ~778,000 residue-specific local quality examples (data points) were generated as the ground truth to evaluate the local predictions of these tools. The true global qualities of the models were also used to evaluate the global quality predictions converted from the local quality predictions.

### Converting local quality scores into one global quality score

Based on the local qualities predicted by the local quality predictor trained on the CASP8 data set, we use a variation of Levitt-Gerstein (LG) score [42] to convert the local quality scores into one global quality score for each individual model:

$$\mathrm{global}=\frac{1}{L}\sum_{i=1}^{L}\frac{1}{1+{\left(\frac{d_{i}}{c}\right)}^{2}}$$

,where *L* is the number of amino acid residues in the protein, *d*_*i*_ is the predicted distance deviation between the position of residue *i* in a model and that in the native structure, and *c* is a constant that was set to 5 in our experiments. This formula was first used by [42] to calculate the similarity score for aligning two protein structures. This formula ensures the global quality remains between (0, 1). The parameter c is a constant, which was set to be 3.5Å for MaxSub score and 5Å for the original LG-score and S-score [42,43]. Another quality prediction method such as ProQ2 [25] also has used similar approaches to convert local scores into global ones.

## Results and discussion

### Benchmarking residue-specific local quality predictions

**Figure 1:**
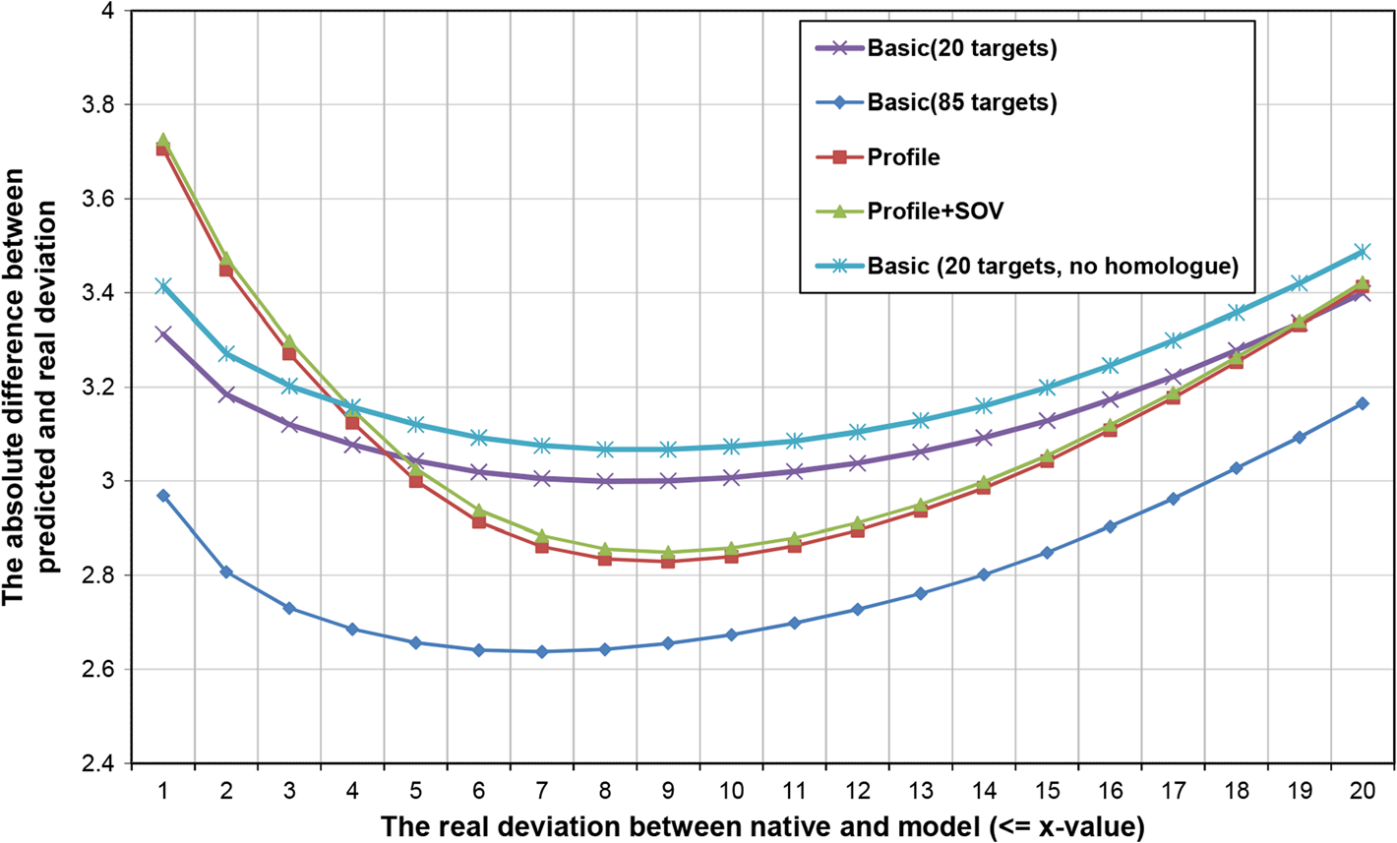
**The evaluation results of residue-specific local quality predictions of single-model local quality QA tools (SMOQ) on CASP9 single-domain proteins.** Basic (20 targets) denotes the SVM model trained using the basic feature set on 20 CASP8 single-domain targets. Basic (85 targets) denotes the SVM model trained using basic feature set on 85 CASP8 single-domain targets. Basic (20 targets, no homologue) denotes the basic model trained on 20 CASP8 single-domain targets, but tested on the CASP9 single-domain targets that are not homologues of CASP8 targets. Profile and profile+SOV denote the two SVM models using profile and profile+SOV feature set that were trained on 20 CASP8 single-domain targets and tested on CASP9 targets without homologue removal. The absolute difference errors of the predictions were plotted against the real distance deviations.

We trained three different SVM models using three different feature sets (“basic”, “profile”, and “profile + SOV score”) extracted from the CASP8 protein models. Using 778,000 CASP9 local quality examples, we benchmarked and compared the performances of the three QA tools (Figure 1). We used the absolute difference between predicted and real deviation between the position of a residue in a model and that of the same residue in the native structure as a metric to evaluate the predictions. We refer to this metric as absolute difference error. According to Figure 1, as the real distance deviation increases, the absolute difference error of predictions of the three tools decreases at first, reaches the minimum and then increases. The best performance of using the basic feature set happened when the real deviation is <= 7Å, where the absolute distance error is ~2.637Å for the basic-feature predictor trained on 85 CASP8 targets.

According to the evaluation results in Figure 1, adding profile and profile+SOV feature did not improve the prediction accuracy over the basic feature set for the cases when real distance deviation is <= 5Å. However, when the real deviation is >5Å, adding profile and profie+SOV starts to improve prediction accuracy. In general, although the basic feature set trained on 85 CASP8 targets performs better than all others SVM models (trained on 20 CASP8 targets) partially because of the larger training data set, a more extensive training on the same large data set is needed in order to more rigorously compare the performance of the feature sets with or without profile and SOV features. The SMOQ tool that we finally released was trained on 85 CASP8 targets using the basic feature set.

We trained the SVM models on CASP8 targets and benchmarked them on CASP9 targets, which contain some homologues of CASP8 targets. Therefore, we also eliminated all the CASP9 targets that are significant homologue to CASP8 targets according to PSI-BLAST comparison and used the remaining CASP9 targets to benchmark the performance of the basic-feature predictor trained on 20 CASP8 targets (see Figure 1). The performance is about 0.1Å worse than without removing homologues.

**Figure 2:**
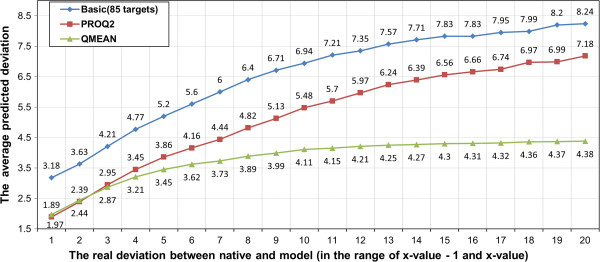
The predicted deviation against real deviation for our basic SVM model and other two local prediction methods (ProQ2 and QMEAN) on 84 CASP9 targets.

The average absolute difference error and average correlation coefficient on all CASP9 examples were reported in Table 1. The average correlation of our basic SVM model trained on 85 CASP8 targets is somewhat lower than ProQ2, but very close to QMEAN. Our basic SVM model performs better than QMEAN in terms of average absolute difference error, but worse than ProQ2. Figure 2 plots the average absolute difference error with respect to different real deviations. Our basic SVM model has higher absolute difference error than ProQ2 or QMEAN for the cases when real deviation is <= 6Å, but for cases whose real deviation is >=7Å, our basic SVM model has lower absolute difference error.

**Table 1:** The average correlation and absolute difference between real and predicted deviation on CASP9 targets for residue-specific quality prediction

	**Avg. correlation**	**Avg. absolute difference error**
*Basic (85 targets)*	0.42	7.09
ProQ2	0.47	6.63
QMEAN	0.43	7.46

**Figure 3:**
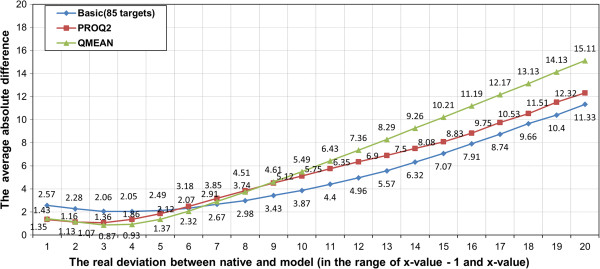
The absolute difference error between real and predicted deviation against real deviation for our basic SVM model and ProQ2 and QMEAN.

Figure 3 shows the relationship between real and predicted distance deviation for basic, ProQ2, and QMEAN. We noticed that QMEAN tends to predict smaller values for deviation when the real deviation actually is large. For example, the predicted deviation remains between 4 to 4.4Å when the real deviation increases from 10 to 20Å. Overall, our SVM model’s performance is somehow comparable to ProQ2 or QMEAN. And our method seems to be complementary with ProQ2 and QMEAN.

### Benchmarking global quality predictions converted from local quality predictions

Based on the residue-specific local quality predictions, we generate absolute global qualities for each TS model. We benchmarked and compared the performance of our local to global quality predictions with the other four single-model global quality prediction tools including ModelEvaluator [18], ProQ [17], ProQ2 [25], and QMEAN [16]. It is worth noting that we only evaluated the performance of these methods on the CASP9 single-domain targets rather than all the kinds of protein targets in order to gauge the accuracy and correctness of our tool. A complete and comprehensive assessment of the other methods can be found in the CASP9 quality assessment paper [44].

Table 2 shows the performances of the QA predictors in terms of average correlation (the average per-target correlation between predicted and real quality scores of the models of each protein target), overall correlation (the correlation between predicted and real quality scores of all the models of all the targets), the average real GDT-TS score of top one models for the targets ranked by each QA predictor, and average loss (the average difference between the GDT-scores of the really best models and those of the top 1 models ranked by each predictor), evaluated on 84 CASP9 single-domain targets. Table 3 reports the performances of the same predictors on eight free modeling (FM) CASP9 single-domain targets.

**Table 2:** The performance of the global quality predictions of our three tools and the other four methods in terms of average correlation, overall correlation, average real GDT-TS score of top 1 models ranked by each method, and average loss of top 1 models ranked by each method, evaluated on 84 CASP9 single-domain targets

	**Avg. correlation**	**Over. correlation**	**Avg. top 1**	**Avg. loss**
*Basic (85 targets)*	**0.737**	0.737	0.588	0.082
*Profile*	**0.708**	0.658	0.589	0.080
*Profile+SOV*	0.696	0.681	0.594	**0.075**
ModelEvaluator	0.636	**0.767**	**0.597**	**0.073**
ProQ	0.494	0.707	0.563	0.110
ProQ2	0.662	**0.787**	**0.607**	**0.066**
QMEAN	**0.733**	**0.803**	**0.594**	0.078

Basic, profile, and profile + SOV are the three single-model local QA tools (SMOQ) presented in this manuscript.

The other four QA predictors are ModelEvaluator (predictor name in CASP9: MULTICOM-NOVEL), ProQ, ProQ2, and QMEAN. Top 3 QA predictors’ performances according to each metric were bolded.

**Table 3:** The performance of the QA predictor in terms of average correlation, overall correlation, average real GDT-TS score of top 1 models ranked by each method, and average loss of top 1 models ranked by each method, evaluated on 8 FM (free modeling) CASP9 single-domain targets

	**Avg. correlation**	**Over. correlation**	**Avg. top 1**	**Avg. loss**
*Basic (85 targets)*	**0.577**	**0.516**	**0.267**	**0.078**
*Profile*	**0.590**	0.427	0.254	0.091
*Profile + SOV*	**0.586**	0.431	**0.267**	**0.078**
M.-NOVEL	0.386	**0.480**	0.235	0.115
ProQ	0.478	0.437	0.266	0.090
ProQ2	0.529	**0.465**	**0.289**	**0.066**
QMEAN	0.507	0.456	0.266	0.090

Top 3 QA predictors’ performances according to each metric were bolded.

It is shown that our predictors using basic/profile features achieved the best or second performances in terms of the average correlation metric (Table 2), which was the official criterion used in the CASP experiment. Our tools also achieved descent, but not the top performance according to other criteria (Table 2). The performance of our tools on the free modeling (or *ab initio*) targets was even better. The models for the free model targets were generated by ab initio protein structure predictors, whose quality was generally much worse than models constructed from known homologous template structures. Thus, it is harder to predict the quality of models of free modeling targets. Table 3 shows that our tool using the basic feature set was constantly ranked within top three. The tool using profile and profile + SOV achieved better performances than the one using basic feature set in terms of the average correlation criteria. Overall, the global quality prediction performance of our tools on the CASP9 single-domain targets is comparable to the best single-model quality predictors.

### An example of local quality predictions

**Figure 4:**
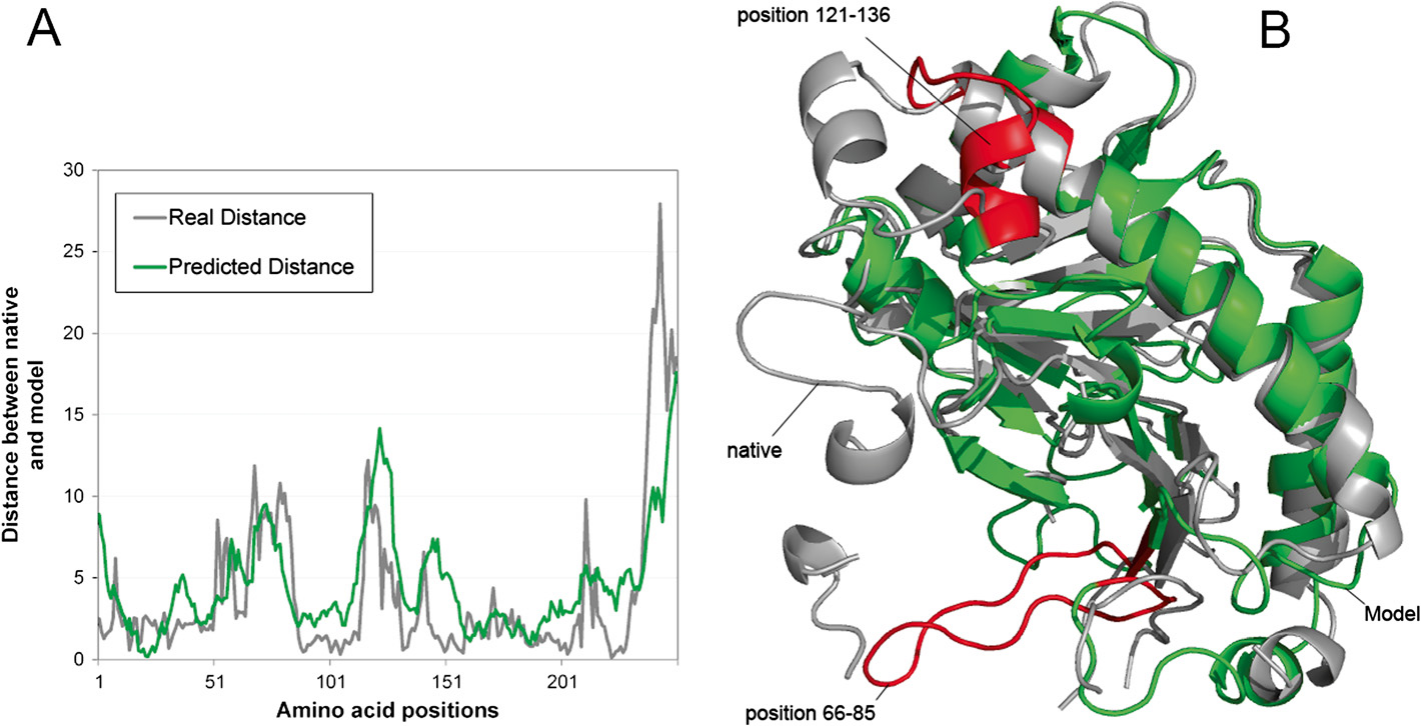
**An example illustrates the real and predicted distances between a model and the native structure.** The model is the first model of the MULTICOM-CLUSTER tertiary structure predictor for CASP9 target T0563. **(A)** The real and predicted distance between the native structure and the model at each amino acid position. **(B)** The superimposition between the model (green and red) and the native structure (grey). Red highlights the two regions where the model has a relatively large deviation compared with the native structure.

Figure 4 illustrates a good example of using our tool based on the basic feature set to predict the local qualities of a model [45] in CASP9. The average difference between real and predicted distance deviation is 2.38Å. This model (green) contains two regions with a relatively large distance deviation with the native structure. One region contains a short helix and the other is a loop. These two regions were highlighted in red in Figure 4 (B). Correspondingly, in Figure 4 (A) the two peaks indicating the larger distance deviation were predicted for these two regions.

## Conclusions

We developed and tested the single-model local quality assessment tools (SMOQ) that can predict the residue-specific absolute local qualities of a single protein model. SMOQ is different from the majority of model quality assessment programs in terms of both methodologies and output. The predicted local qualities were also converted into one single score to predict the global quality of a model. The SMOQ tools were rigorously tested on a large benchmark and yielded a performance comparable to other leading methods. However, in this work, we only used single-domain CASP8 targets for training. In the future, we plan to include multi-domain targets by cutting a whole multi-domain model into individual domains and only aligning each domain with its native structure to generate real local quality scores for training. Another future work is to test other functions of converting local scores into global ones. Overall, we believe that SMOQ is a useful tool for both protein tertiary structure prediction and protein model quality assessment.

## Availability and requirements

**Project name:** SMOQ

**Project homepage:**http://sysbio.rnet.missouri.edu/multicom_toolbox/

**Operating systems:** Linux

**Programming language:** Perl

**Other requirements:** no

**License:** Free academic usage

**Any restrictions to use by non-academics:** For non-academic use, please contact the corresponding author for permission

## Competing interests

The authors declare that they have no competing interests.

## Authors’ contributions

JC conceived the project. JC, ZW, RC designed the project. ZW, RC, and YW implemented and tested the tool. RC, ZW, JC prepared the software package. ZW, RC, JC wrote the manuscript. All the authors read and approved the manuscript.
